# Supreme™ laryngeal mask airway insertion requires a lower concentration of sevoflurane than ProSeal™ laryngeal mask airway insertion during target-controlled remifentanil infusion: a prospective randomised controlled study

**DOI:** 10.1186/s12871-019-0921-5

**Published:** 2020-01-07

**Authors:** Cristina Monteserín-Matesanz, Tatiana González, María José Anadón-Baselga, Matilde Zaballos

**Affiliations:** 1 Anaesthesia department, Hospital General Universitario Gregorio Marañón, C/ Doctor Esquerdo, N° 46, 28007 Madrid, Spain; 2 Department of Legal Medicine, Psychiatry and Pathology Universidad Complutense, Madrid, Spain

**Keywords:** End-tidal sevoflurane concentration, Supraglottic airway devices, Remifentanil effect-site concentration, Laryngeal mask airway supreme, Laryngeal mask airway Proseal

## Abstract

**Background:**

ProSeal (PLMA) and Supreme (SLMA) laryngeal mask airways are effective ventilator devices with distinctive designs that may require different anaesthetics for insertion. Sevoflurane induction provides acceptable conditions for laryngeal mask insertion, and remifentanil significantly decreases the minimum alveolar concentration of sevoflurane required for that insertion. The study aimed to evaluate the optimal end-tidal (ET) sevoflurane concentration for successful insertion of PLMA versus SLMA in patients receiving a remifentanil infusion without a neuromuscular blocking agent.

**Methods:**

Altogether, 45 patients ASA (American Society Anaesthesiologists) physical status I–II, aged 18–60 years were scheduled for elective ambulatory surgery. Exclusion criteria were a difficult airway, recent respiratory infection, reactive airway, obstructive sleep apnoea syndrome, gastric aspiration’s risk factors, pregnancy, and lactation. Patients were randomly allocated to receive the SLMA or the PLMA. Sevoflurane induction with co-administration of remifentanil was performed at an effect-site concentration of 4 ng mL^− 1^. ET_50_ was calculated with a modified Dixon’s up-and-down method (starting at 2.5% in steps of 0.5%). Predetermined sevoflurane concentration was kept constant during the 10 min before LMA insertion. Patient’s response to LMA insertion was classified as “movement” or “no movement”. Sevoflurane ET_50_ was determined as the midpoint concentration of all the independent pairs that manifested crossover from “movement” to “no movement”.

**Results:**

The ET_50_ sevoflurane concentration co-administered with remifentanil required for PLMA insertion was 1.20 ± 0.41% (95% confidence interval 0.76 to 1.63%). For SLMA insertion, it was 0.55 ± 0.38% (95% confidence interval 0.14 to 0.95%) (*p* = 0.019).

**Conclusions:**

The end-tidal sevoflurane concentration with co-administered remifentanil required to allow insertion of the SLMA was 54% lower than that needed for inserting the PLMA.

**Trial registration:**

Clinicaltrials.gov identifier: NCT03003377. Retrospectively registered. Date of registration: December 28, 2016.

## Background

The ProSeal™ laryngeal mask airway (PLMA) (Teleflex, Teleflex Medical Europe, Westmeath, Ireland) was the first second-generation reusable device designed to separate the gastrointestinal and respiratory tracts. It exhibited safety and efficacy as an instrument for providing adequate ventilation during general anaesthesia even for advanced clinical uses [1]. The Supreme™ laryngeal mask airway (SLMA) (Teleflex, Teleflex Medical Europe, Westmeath, Ireland) was developed in 2007 as a modified single-use second-generation device that combines the design of the Fastrach™ laryngeal mask airway (Teleflex, Teleflex Medical Europe, Westmeath, Ireland) and the PLMA. The gastric tube of the SLMA is incorporated within an oval airway tube designed to match the shape of the mouth and oropharyngeal inlet and facilitate its insertion [2].

The two devices have differences in their structure, design, and components, which means different compression in the pharyngeal structures during the placement phase and thus influencing the anaesthetic requirements. The anaesthetic strategy commonly used for insertion of LMAs relies on administration of an intravenous (propofol) or a volatile (sevoflurane) induction agent with or without a co-induction agent such as an opioid (fentanyl, alfentanil, remifentanil), midazolam, or lidocaine [3–6]. The use of a co-induction agent could facilitate and significantly reduce the dose of induction agent required for LMA insertion.

Previous studies have compared the effectiveness and safety of the PLMA and SLMA in different clinical scenarios, showing differences regarding the oropharyngeal leak pressure, success rate, insertion time, and airway complications [7–10]. In contrast, little information is available regarding the optimal end-tidal sevoflurane concentration when used for co-induction with remifentanil to ensure successful LMA insertion.

Because of the features of the SLMA and its ease of insertion, we hypothesised that the predicted end-tidal (ET) concentration of sevoflurane during co-induction with a target-controlled infusion of remifentanil (4 ng/ml) without neuromuscular blocking drugs in adult patients would be lower than that for PLMA.

## Methods

### Study design

We conducted a single-centre, double-blind, randomised controlled trial registered at www.clinicaltrials.gov (number NCT03003377). Ethical approval for this study (Ethical Committee code FIBHGM-ECNC002–2013) was provided by the Ethics Committee of Hospital General Universitario Gregorio Marañón, Madrid, Spain (Chairman Dr. Fernando Díaz Otero) on 12 June 2013. Patient were consecutively enrolled in the study from November 2014, to October 2015. This study is reported in accordance with the CONSORT-Statement.

### Participants

We enrolled 55 patients (ASA physical class I–II, aged 18–60 years) scheduled for elective ambulatory surgery under general anaesthesia and in whom the use of a supraglottic airway was indicated. We excluded patients with more than three criteria for a difficult airway [Mallampati III–IV, thyromental distance < 6 cm, limited mouth opening (≤3 cm), cervical spine disease], increased risk of aspiration, recent upper respiratory tract infection, pregnancy, lactation, body mass index exceeding 35 kg.m^2^ and/or patient refusal to participate in the study. Patients in psychiatric treatment, abuse of alcohol or use any medication that could interfere with the study were also excluded. All participants provided written informed consent prior to study entry.

### Randomisation and blinding

Participants were randomly assigned to the PLMA or SLMA group according to a computer-generated block randomisation sequence using the Research-Randomizer program, version 4.0 (http://www.randomizer.org/). The sequence was stored in sealed opaque envelopes kept by the study coordinator (MZ). A single study investigator (CM) had access to the randomisation code and opened the envelope before the scheduled case at which time the patient was assigned to his or her study group.

### Intervention

Routine monitoring, including pulse oximetry, heart rate, and non-invasive arterial blood pressure, were applied (Datex-Ohmeda Cardiocap™/5, Louisville, CO, USA). In addition to standard monitoring, the Bispectral Index (BIS VISTA™ Monitoring System, Aspect Medical Systems, Inc., Mansfield, MA, USA) was used in all patients. Inhaled and exhaled concentrations of O_2_, CO_2_, and sevoflurane were monitored breath by breath (Datex-Ohmeda Cardiocap™/5).

The patients were given midazolam 1 mg IV 20 min before anaesthesia induction. All patients were preoxygenated with 100% oxygen for 3 min. The anaesthetic circuit was then filled with 5% sevoflurane at a fresh gas flow of 6 l min^− 1^ for 3 min. Inhalational anaesthesia started with simultaneous target-controlled infusion (TCI) of remifentanil with the pharmacokinetic model of Minto through a commercial TCI pump (Alaris® PK, Cardinal Health, 1180 Rolle, Switzerland) adjusted to an effect-site concentration of 4 ng ml^− 1^ [11].

Patients were manually ventilated, if needed, to maintain normal PCO_2_ values (35–40 mmHg). After loss of consciousness, the inspired sevoflurane concentration was adjusted in each participant to obtain the predetermined ET concentration of sevoflurane using the modified sequential Dixon’s up-and-down methodology [12]. Thus, each patient’s response determined the sevoflurane concentration used in the next patient. The first patient’s predetermined ET concentration of sevoflurane was 2.5% delivered in steps of 0.5% (although below the limit of sevoflurane 0.5%, the step size was 0.1%). In each participant, the predetermined sevoflurane concentration was maintained for more than 10 min to ensure equilibration between the alveolar gas tension, blood, and cerebral tissue before attempting any device insertion. An anaesthesiologist (MZ) experienced in the use of LMA (> 200 cases) inserted the randomly allocated device following the manufacturer’s recommendations without using neuromuscular blocking agents. The digital insertion technique was performed with the PLMA. The LMA size was chosen according to the patient’s sex (size 4 for women, size 5 for men), although size 3 was inserted for subjects weighing ≤50 kg. However, a change in the size or in the LMA device was permitted according to the judgement of the attending anaesthetist. The LMA cuff was inflated to 60 cm H_2_O after insertion. Once stable ventilation with oxygen in air was established, the oropharyngeal leak pressure (OLP) was measured closing the expiratory valve to 40 cm H_2_O and maintaining fresh gas flow at 3 l min^− 1^. The rising pressure within the system was measured with a pressure gauge and was allowed to increase until it reached equilibration, which was considered the OLP.

The participant’s response to the LMA insertion was classified as “failure” or “success” by the surgeon and/or nurse, who were blinded to the sevoflurane concentration. Failure was defined as the presence of coughing, bucking, laryngospasm, or gross purposeful withdrawal movement of the extremities within 1 min of insertion. The presence of laryngospasm should be confirmed by the anesthesiologist performing the LMA insertion. The absence of verbal contact before SADs insertion were classified as ‘movement’. The presence of minor finger movement or hiccup was not classified as failure. Jaw relaxation was evaluated and graded according to Muzi’s score [13]—that is, 1: fully relaxed, 2: mild resistance, 3: tight but could be opened, 4: closed requiring a dose of propofol. To guarantee patient comfort, an intravenous bolus dose of propofol 1–2 mg kg^− 1^ was administered to each subject experiencing a positive response during LMA insertion. A single measurement was obtained from each participant.

Haemodynamic data, respiratory parameters, and BIS values were recorded at baseline immediately before LMA insertion and 1 and 6 min after LMA insertion. Hypotension was defined as mean arterial pressure < 50 mmHg and was treated with ephedrine 3 mg. Bradycardia was defined as heart rate < 45 bpm and was treated with atropine 0.1 mg kg^− 1^.

All study subjects were interviewed in the recovery room to assess memory recall by a blinded observer.

### Statistical analysis

The sevoflurane ET_50_ co-administered with remifentanil required for PLMA and SLMA insertions was determined by calculating the midpoint concentration of all the independent pairs of patients who manifested crossover from a movement response to a non-movement response. The standard deviation of the sevoflurane ET_50_ represented the standard deviation of the crossover midpoint of each group.

Dose–response curves were assessed to determine the probability of no movement relative to the sevoflurane concentration and to obtain a sevoflurane concentration where 50% (ET_50_) and 95% (ET_95_) of the device attempts were successful in both groups and the maximum likelihood estimators of the model parameters. Goodness of fit was obtained using logistic regression curves [14].

Sevoflurane ET_50_ values in the PLMA and the SLMA groups were compared using Student’s t-test. Haemodynamic data and the BIS value were compared by repeated measures analysis of variance. The 휒^2^ test, with Fisher’s exact probability test, when appropriate, was used to compare jaw relaxation. The OLP was compared using the unpaired Student’s t-test.

A value of *P* < 0.05 was considered to indicate statistical significance.

Statistical analyses were performed using SPSS 22.0 software for Windows (IBM Corp., Armonk, NY, USA).

### Simple size calculation

We applied the Dixon approach for simple size calculation for the up-and-down method design. In similar studies in the field of anaesthesia, the number of crossovers varies between six and eight with six crossovers being most common. For this study’s purposes, the allocation sequence continued until the six crossovers points from “failure” to “success” were obtained in each group [12, 15].

## Results

**Fig. 1:**
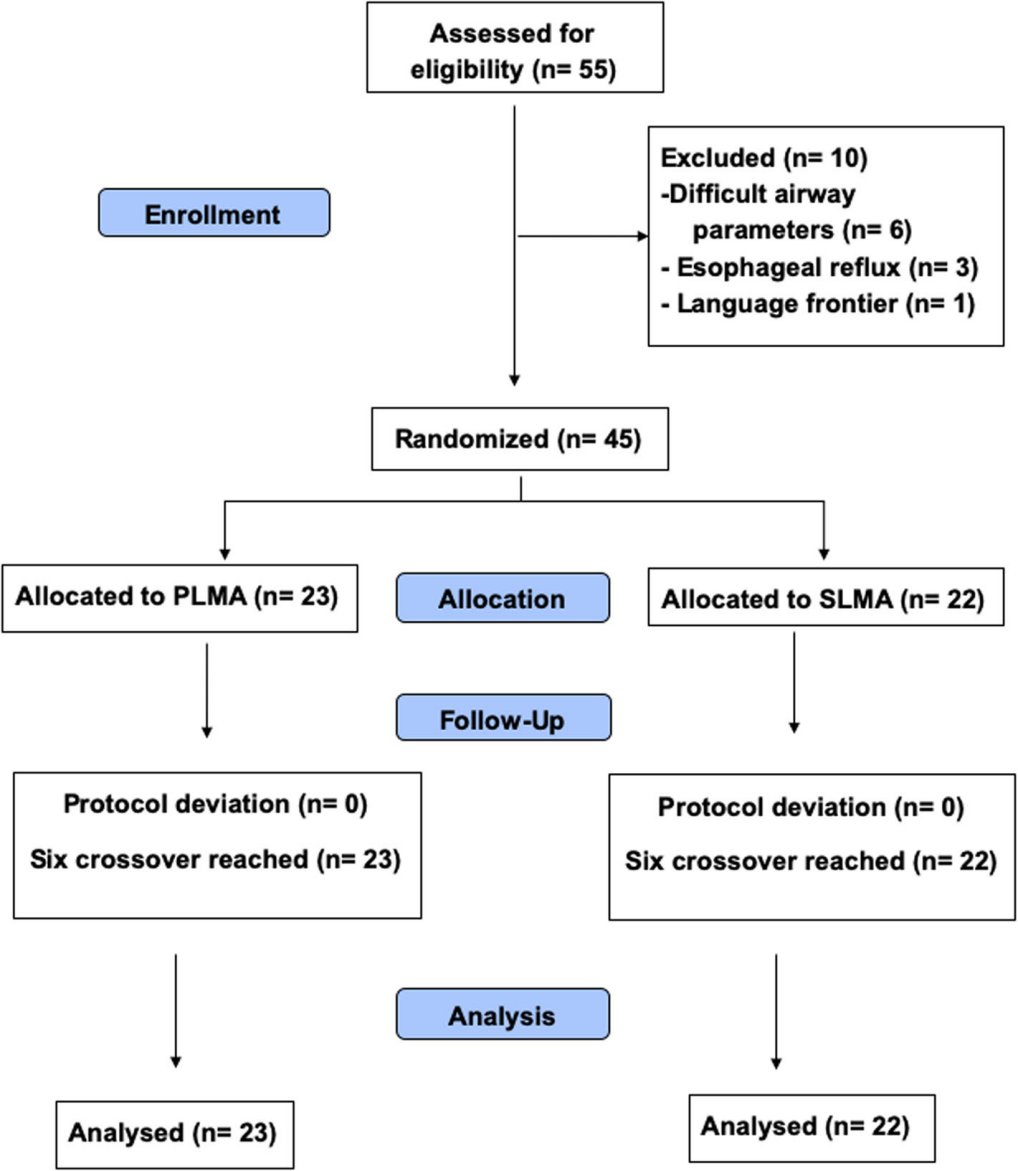
Flow-diagram of patient progress through the phases of the trial. Patients were recruited until a sample size of seven crossovers was reached in each group

Participants’ flow during the study is shown in Fig. 1. Forty-five subjects were randomised to either the PLMA (*n* = 23) or the SLMA (*n* = 22) group. There were no significant differences in terms of patients’ characteristics although general surgery was performed more frequently in the PLMA group and vascular surgery in the SLMA group (Table 1).

**Table 1:** Demographic data of patients and surgical procedures

	PLMA (*n* = 23)	SLMA (*n* = 22)
Patients	23	22
Age (yr)	43 ± 13	46 ± 11
Female/Male	15/8	15/7
Weight (kg)	72 ± 13	75 ± 16
Height (cm)	169 ± 8	168 ± 9
BMI	24.98 ± 3.61	26.32 ± 4.63
Mallampati classification		
I	12	11
II	10	7
III	1	4
ASA I / ASA II	13/10	11/11
Surgical procedure*		
Vascular (varicose veins)	6	12
Orthopaedic	3	3
General	14	7

Data are expressed as mean ± SD or number.

*PLMA* Laryngeal mask airway ProSeal™, *SLMA* Laryngeal mask airway Supreme™, *BMI* Body mass index, *ASA* American Society of Anaesthesiologists´ physical status, *SD* Standard deviation.

* *p* = 0.04 for surgical procedure

**Fig. 2:**
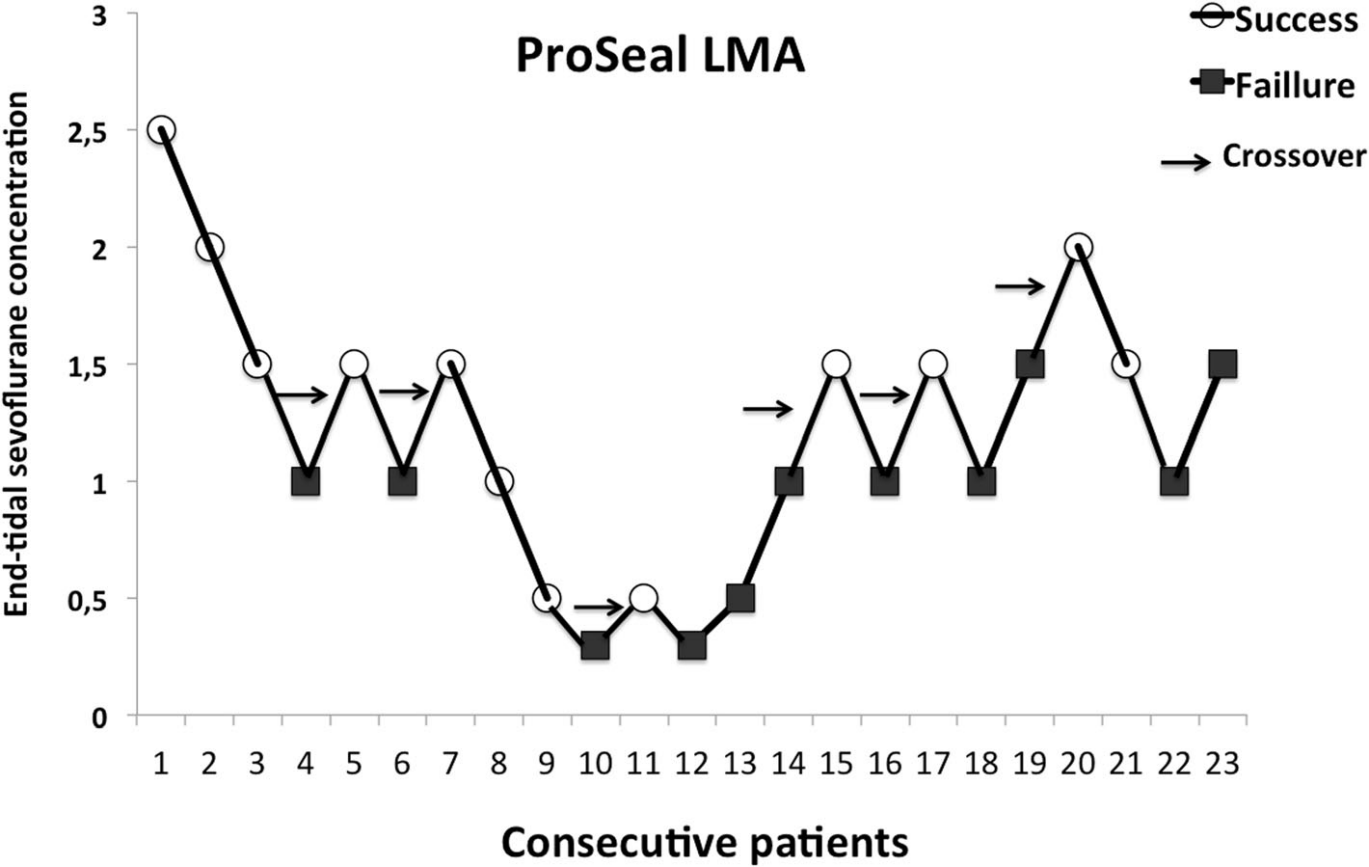
Patients’ responses to Laryngeal Mask Airway ProSeal™ insertion. Arrows indicate the midpoint of the effect-site concentration of all independent pairs of patients involving crossover from device insertion failure to successful Laryngeal Mask Airway Airway ProSeal™ insertion

**Fig. 3:**
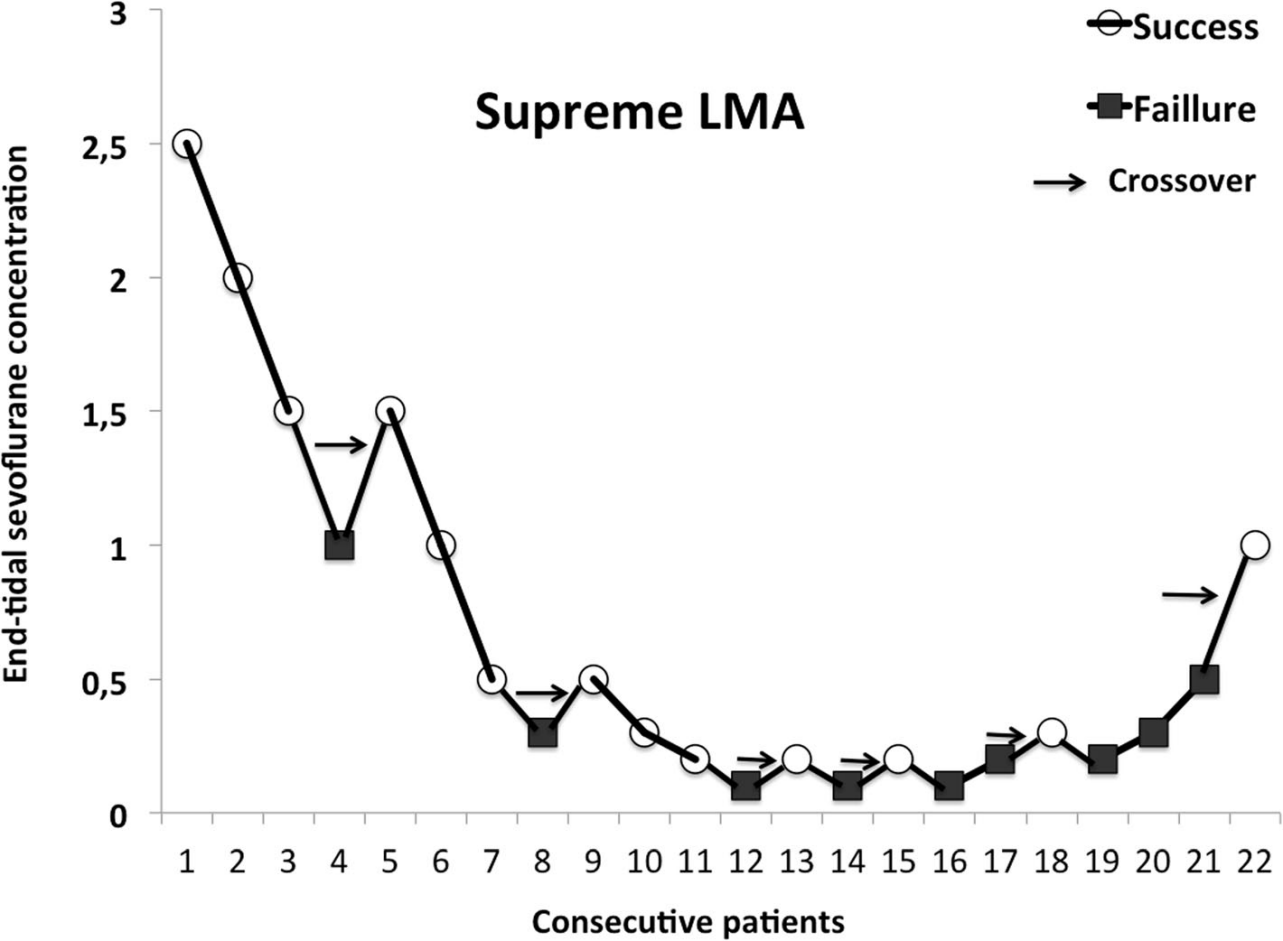
Patients’ responses to Laryngeal Mask Airway Supreme™ insertion in Arrows indicate the midpoint of the effect-site concentration of all independent pairs of patients involving crossover from device insertion failure to successful Laryngeal Mask Airway Supreme™ insertion

**Fig. 4:**
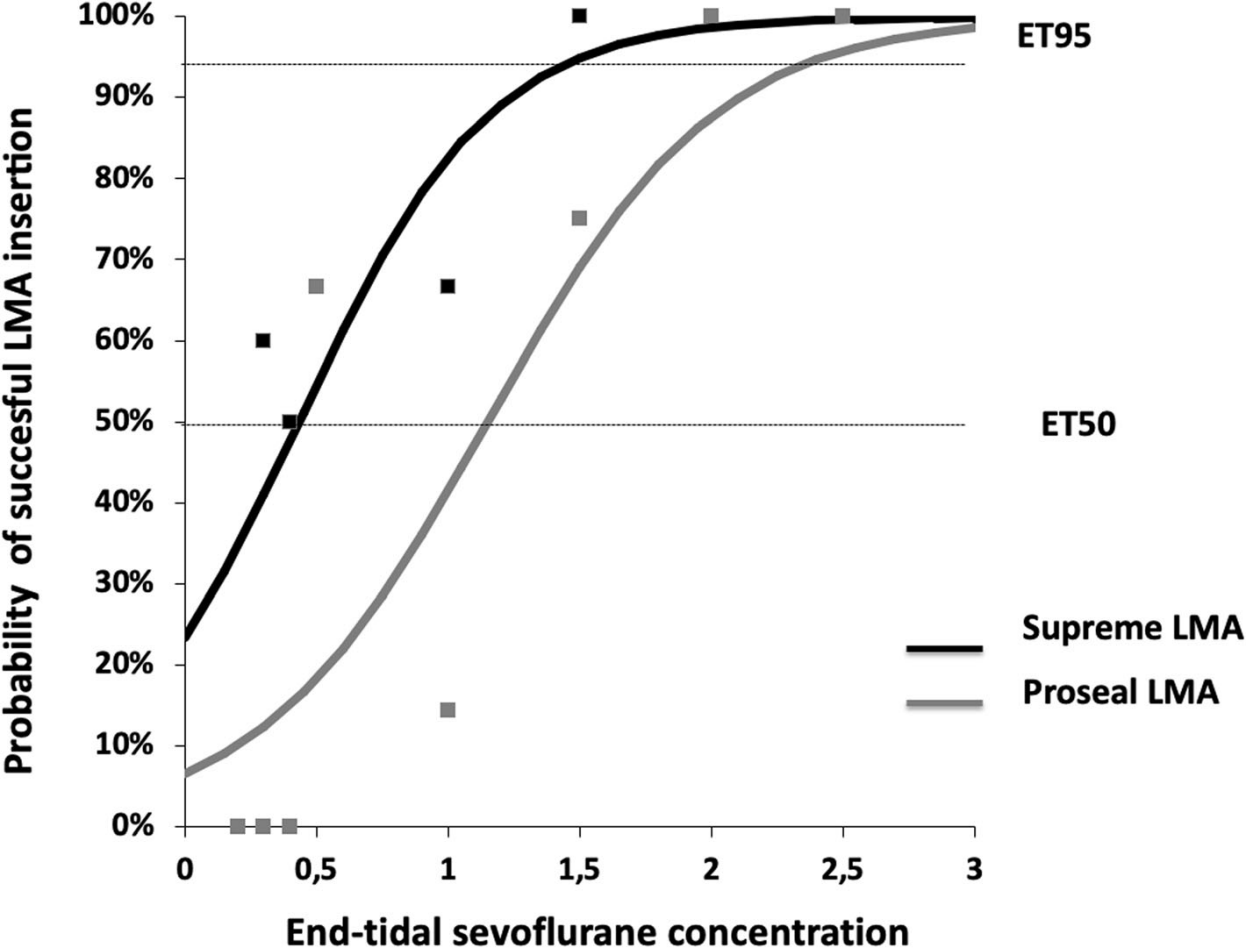
Dose-response curves plotted from logistic analysis of individual end-tidal sevoflurane concentrations and the respective reactions to PLMA or SLMA insertion. ET50 in PLMA group: 1.15%; ET50 in SLMA group: 0.43%; ET95 in PLMA group: 2.43%; ET95 in SLMA group: 1.50%

Individual dose–response data obtained by Dixon’s up-and-down method are shown in Fig. 2 (PLMA) and Fig. 3 (SLMA). The predicted ET_50_ of sevoflurane was significantly higher for successful PLMA insertion (1.20 ± 0.41%[95% CI 0.76–1.63]) than for SLMA insertion (0.55 ± 0.38% [95% CI 0.14–0.95] (*p* = 0.019). Using logistic regression curves, the ET_50_ and the ET_95_ of sevoflurane required for PLMA insertion were 1.15% (95% CI 0.57–2.33) and 2.43% (95% CI 1.10–5.34), respectively. For SLMA insertion, they were 0.43% (95% CI 0.02–7.76) and 1.50% (95% CI 0.55–4.08) respectively (Fig. 4). Table 2 presents the estimated values from the logistic and goodness-of-fit analyses.

**Table 2:** Estimated values of the of the logit coefficients

	PLMA	SLMA
ET-50% LMA (CI)	1.15 (0.57–2,33)	0.43 (0.02–7.76)
ET 95% LMA (CI)	2.43(1.10–5.34)	1.50(0.55–4.08)
B0	−2.647	−1.188
B1	2.304	2.749
*p* Value	0.106	0.676
Goodness of fit chi-squared	7.634	3.170

*CI*: 95% confidence interval.

p/(1 − p) = B_0_ + B_1X_.

B0 = intercept; B1 = slope; X = end-tidal concentration (%).

In two participants in the PLMA group, we changed the size of the LMA (size 4 to a size 3). In three patients in the SLMA group, we changed the SLMA to a PLMA because of inadequate ventilation. Overall, we found a higher incidence of patients with resistance to jaw relaxation and requiring propofol in the PLMA group but without statistical significance (*p* = 0.30) (Table 3).

**Table 3:** Assessment of jaw relaxation according to Muzi score

	PLMA (*n* = 23)	SLMA (*n* = 22)
Fully relaxed.	12	13
Mild resistance.	2	3
Resistance but could be opened.	2	4
Resistance requiring a dose of propofol (mg)	7 111 ± 12	2 110 ± 14

Data are expressed as number of patients or mean ± SD.

Baseline BIS and haemodynamic data did not differ between the two groups (Table 4). In both groups, the heart rate, systolic and diastolic pressures, and BIS significantly decreased relative to baseline values. The number of subjects who required atropine or ephedrine did not differ significantly.

**Table 4:** Haemodynamic and BIS data at different times in the two groups

	PLMA (*n* = 23)	SLMA (*n* = 22)
Systolic arterial pressure		
Baseline	138 ± 16	135 ± 20
Before insertion	98 ± 13 [29%]	96 ± 15 [29%]
1st min post-insertion*	101 ± 13 [27%]	108 ± 17 [20%]
6th min post-insertion*	98 ± 13 [29%]	97 ± 14 [28%]
Diastolic arterial pressure		
Baseline	80 ± 12	79 ± 11
Before insertion*	56 ± 9 [30%]	53 ± 9 [33%]
1st min post-insertion*	58 ± 13 [30%]	60 ± 12 [24%]
6th min post-insertion*	55 ± 11 [31%]	57 ± 8 [28%]
Heart rate		
Baseline	73 ± 17	73 ± 13
Before insertion*	56 ± 11 [23%]	54 ± 9 [26%]
1st min post-insertion*	57 ± 10 [22%]	59 ± 11 [19%]
6th min post-insertion*	56 ± 12 [23%]	60 ± 11 [18%]
BIS value		
Baseline	95 ± 4	96 ± 5
Before insertion*†	60 ± 8 [37%]	63 ± 9 [35%]
1st min post-insertion*†	56 ± 13 [41%]	64 ± 14 [34%]
6th min post-insertion*†	41 ± 15 [57%]	43 ± 16 [55%]

Data are expressed as mean ± SD [% difference from baseline].

*PLMA* Laryngeal mask airway ProSeal™, *SLMA* Laryngeal mask airway Supreme™, *BIS* Bispectral index, *SD* Standard deviation.

* *p* < 0.05 for significant differences from baseline (difference within the group) by repeated measures ANOVA

† *p* < 0.05 for significant differences between the PLMA and the SLMA groups by repeated measures ANOVA.

The BIS value significantly differed between the two groups, being higher in the SLMA group than in the PLMA group (Table 4). Nevertheless, no participant manifested intraoperative recall during recovery.

No episodes of laryngospasm were described, although three subjects experienced peripheral oxygen desaturation of < 90% during LMA insertion (one patient in the PLMA group and two in the SLMA group). In all cases, it recovered after the LMA was in place and working.

The mean OLP was higher in the PLMA group (24.42 cm ± 4.9 cm H_2_O) than in the SLMA group (22.55 cm ± 3.97 cm H_2_O), but the difference was not statistically significant.

## Discussion

To our knowledge, this is the first randomised study designed to compare the ET_50_ of the sevoflurane concentration during co-induction with remifentanil TCI at 4 ng mL^− 1^, which is required for successful insertion of the PLMA and SLMA in adult patients. The results of the present study show that the SLMA can be inserted at a lower sevoflurane concentration than that required for the PLMA. (The ET_50_ value of sevoflurane for SLMA insertion was 54% less than that for PLMA.)

Previous reports found that insertion of a PLMA may take longer and require more attempts than the SLMA requires [7, 9]. This finding might be attributed to several factors such as the insertion technique of each LMA because of the design variations between the devices. The anatomically shaped airway tube and thin wedge-shaped leading edge of the SLMA have been purported to permit smoother, successful insertion with a simple circular movement. In contrast, during placement, the posterior aspect of the PLMA is pressed up against the hard palate with a finger maintaining a constant backward pressure to facilitate its passage around the posterior pharyngeal wall. These differences in insertion technique can affect the pattern and intensity of stimulation and thus the anaesthetic needs of the devices. A greater proportion of patients in the PLMA group showed resistance regarding relaxation of the jaw.

Kodaka et al. [3] compared the ET_50_ of sevoflurane required for CLMA and PLMA insertions and observed that the ET_50_ of sevoflurane for PLMA placement was 20% higher (2.82 ± 0.45%) than that for CLMA placement (2.36 ± 0.22%). Our results show that adding remifentanil induces a significant reduction in the sevoflurane requirement. In fact, the ET_50_ of sevoflurane for PLMA placement (1.20 ± 0.41%) was nearly 60% less than that reported by Kodaka [3]. Zaballos et al. [4], using the up-and-down method, showed that the ET_50_ of sevoflurane for SLMA placement was 3.03 ± 0.75% in patients premedicated with 1 mg of midazolam. The results of the present study showed an even greater reduction of the sevoflurane concentration (82%) needed for SLMA insertion (0.55 ± 0.38%) when remifentanil was added.

Adding a potent, short-acting opioid such as remifentanil during sevoflurane inhalation induction has been reported to improve conditions for LMA insertion or tracheal intubation, decreasing the incidence of excitatory movements during induction [5, 6, 16, 17]. This effect of remifentanil may be due to blockade of afferent nerve impulses resulting from stimulation of the laryngopharynx during LMA insertion and cuff inflation. Although adequate induction for LMA placement can be achieved using sevoflurane alone, an opioid analgesic is commonly co-administered to increase synergistically the clinical anaesthetic level, thereby facilitating the LMA placement [5, 17].

Other studies are in agreement that the ET_50_ of sevoflurane needed for PLMA insertion is higher than that required for other first-generation devices. To our knowledge, however, no studies have compared the sevoflurane requirement for PLMA insertion with that for other second-generation devices [3, 18].

>BIS values were significantly higher in the SLMA group, which is consistent with the lower sevoflurane administration in this group. However, no patient reported recall when questioned in the recovery room. Manyam et al. [19] investigated the impact on BIS values when adding remifentanil to sevoflurane in doses sufficient to change the clinical level of sedation. Although clinical sedation increased significantly with the addition of remifentanil to a sevoflurane anaesthetic, the BIS was insensitive to the change in the clinical level of sedation. The authors suggested that during sevoflurane-remifentanil anaesthesia, targeting a BIS < 60 may result in an excessively deep anaesthetic state.

Our data support this finding as the addition of remifentanil at an effect-site concentration of 4 ng mL^− 1^ to the different sevoflurane concentrations generated a mean BIS value of 61 ± 9 before insertion of the LMA without significant differences between subjects who showed a “movement” response and those who did not (64 ± 8 vs. 60 ± 10, respectively; *p* = 0.16).

Oropharyngeal leak pressure was higher in the PLMA group, which is consistent with the results of previous publications [7]. This finding may be related to a deeper anaesthetic plane, which could influence the tone of the pharyngeal muscles.

This study had some limitations. First, our main objective was to determine the sevoflurane ET_50_ for co-administration with remifentanil that was required for successful insertion of the PLMA and the SLMA according to Dixon’s up-and-down method. A minimum amount of time is required to guarantee drug concentration equilibration between phases (10 min in the present study). This prolonged time is not representative of the clinical experience. Second, according to Dixon’s design, the sample size is limited when a specific number of crossovers (4–10) between up-and-down steps have been achieved. It is typically limited to 20–40 patients. Because the effect of varying the number of crossovers can induce bias in the estimation, there is agreement that six crossovers are sufficient [15, 20]. Third, an expert anaesthesiologist on regular use of supraglottic airway devices in clinical practice inserted all the devices. Our results therefore cannot be extrapolated to the insertion of LMAs by novice users.

## Conclusion

The end-tidal sevoflurane concentration during remifentanil co-administration needed to allow insertion of the SLMA is 54% lower than that needed for insertion of the PLMA. Both devices are effective for applying positive-pressure ventilation to patients undergoing ambulatory surgery with few adverse effects.

## Data Availability

The datasets used and/or analyzed during the current study are available from the corresponding author on reasonable request.

## Abbreviations

ASA: American society anesthesiologist
BIS: Bispectral index
ET: End-tidal
ET_50_: ET concentration in 50%
ET_95_: ET concentration in 95%
MAC: Minimal alveolar concentration
OLP: Oropharyngeal leak pressure
PLMA: ProSeal laryngeal mask airway
SLMA: Supreme laryngeal mask airway
TCI: Target controlled infusion
